# Discovery of metabolite biomarkers for cerebral ischemia-reperfusion injury based on metabolomics: neurotoxic mechanism and functional validation of a flavopiridol -related metabolite

**DOI:** 10.3389/fmolb.2026.1752337

**Published:** 2026-10-01

**Authors:** Zhi-guang Song, Yu-chen Liu, Zhen-lin Zhong, Shu-ting Yin, Si-si Dai, Yi-di Zeng, Cai-xing Zheng, Si-yuan Chen, Ji-yong Liu, Yan-song Liu, Si-min Peng, Shi-min Tan, Qian-ru Zeng, Hua Li, Wang-hua Liu

**Affiliations:** 1 Key Laboratory of TCM Diagnostics of Hunan Province, Hunan University of Chinese Medicine, Changsha, Hunan, China; 2 Hunan Engineering Technology Research Center for Medicinal and Functional Food, Changsha, Hunan, China; 3 Key Laboratory of TCM Heart and Lung Syndrome Differentiation and Medicated Diet and Dietotherapy, Changsha, Hunan, China

**Keywords:** cerebral ischemia-reperfusion injury, flavopiridol, molecular docking, molecular dynamics simulation, network toxicology, untargeted metabolomics

## Abstract

**Objective:**

To investigate the role and potential mechanism of metabolite alterations during cerebral ischemia-reperfusion injury (CIRI) and to explore novel candidate molecular markers for the diagnosis of this disease.

**Methods:**

A rat model of middle cerebral artery occlusion was established using the modified Longa EZ intraluminal suture method. The infarct status was comprehensively evaluated by neurological deficit score (mNSS), TTC staining, HE staining, and Nissl staining. Serum metabolite changes after CIRI were detected using non-targeted metabolomics, and the potential toxic effects of significantly altered metabolites were predicted using the ProTox tool. Subsequently, bioinformatics analysis was employed to identify core targets and key pathways associated with the metabolite of interest, followed by molecular docking and molecular dynamics simulations to predict their interactions.

**Results:**

Metabolomic analysis identified 41 significantly upregulated and 232 downregulated metabolites. Among the significantly altered metabolites, a flavopiridol‐related metabolite (FRM) exhibited potential neurotoxic characteristics. A total of 193 CIRI-related core targets were associated with this metabolite, from which 10 core targets and 15 key pathways were further screened. ROC analysis suggested that this metabolite possessed good discriminatory ability (AUC = 0.94). Molecular docking results showed binding energies of less than −7 kcal/mol for all core targets. A 100 ns molecular dynamics simulation indicated that the complex formed by the FRM and SRC remained structurally stable, as evidenced by balanced RMSD, Rg, SASA, and P-L-RMSD values. The calculated binding free energy was −32.48 kcal/mol, with key residues LEU-393, MET-341, and GLU-339 contributing significantly to the interaction, suggesting potential biological activity.

**Conclusion:**

This study reveals a complex metabolic reprogramming phenomenon during CIRI and preliminarily identifies a FRM as a potential candidate molecular marker and possible interventional target, providing a new theoretical basis and directions for future research on the diagnosis and treatment of CIRI.

## 1 Introduction

According to the latest estimates from the Global Burden of Disease Study (GBD) 2021, stroke remains a major global health threat, ranking as the third leading cause of death and the fourth leading cause of disability-adjusted life years (DALYs) worldwide. In 2021, stroke was responsible for approximately 7.3 million deaths and 160.5 million DALYs globally. Between 1990 and 2021, the number of incident strokes, stroke-related deaths, and overall disability burden increased significantly, with the annual number of new strokes rising by 70% and deaths increasing by 44% (Feigin et al., 2024). Projections from the World Stroke Organization (WSO) and the Lancet Neurology Commission, as highlighted by the Global Stroke Action Coalition, indicate that without effective intervention, annual global stroke deaths could increase by 50% over the next 25 years (Feigin et al., 2023). Furthermore, the economic impact of stroke is substantial, with annual global economic losses estimated at US$1.6 trillion, a figure that is expected to rise significantly by 2050. Ischemic stroke is the most common type, accounting for approximately 65.3% of all incident strokes globally. In modern stroke care, timely restoration of blood flow to the ischemic area is a core strategy for improving patient outcomes. However, research indicates (Wicha et al., 2020; Ruan et al., 2023) that reperfusion can trigger a complex pathological cascade involving oxidative stress (e.g., reactive oxygen species explosion), calcium overload, and inflammatory responses, leading to secondary brain damage known as cerebral ischemia-reperfusion injury (CIRI).

Immediately after reperfusion, excitotoxicity mediated by glutamate and other amino acid compounds triggers a cascade of pathological events, including neuroinflammation, oxidative stress, increased blood–brain barrier permeability, cerebral vasodilation, and neuronal apoptosis (Rothman and Olney, 1986). These processes disrupt neurotransmission homeostasis and contribute to neuronal injury or death. Lipid peroxidation (LPO), driven by free radicals attacking polyunsaturated fatty acids (PUFAs) on cell membranes and proteins, is recognized as a central mechanism in numerous oxidative stress-related diseases (Yin and Porter, 2005) and is closely associated with both apoptotic and necrotic cell death (Tyurin et al., 2007). Moreover, metabolites derived from arachidonic acid—such as prostaglandin E2 (PGE2)-exhibit dual roles in inflammation and neurotoxicity, serving as key inflammatory mediators in conditions including osteoarthritis, allergic asthma, rheumatoid arthritis, and stroke (Ricciotti and FitzGerald, 2011; Park et al., 2006). Collectively, these toxic factors contribute to a “second wave of injury,” resulting in delayed neuronal death and exacerbating cerebral ischemia-reperfusion injury (Rawlinson et al., 2020; Bersano and Gatti, 2023). Given these mechanisms, there is an urgent need to develop novel neuroprotective strategies. Accordingly, this study aims to investigate dynamic changes in serum endogenous metabolites following CIRI and to identify potential specific molecular biomarkers for early diagnosis, thereby providing new insights for early intervention.

Network toxicology, a concept pioneered by Academician Chang-xiao Liu (Fan et al., 2011; Li et al., 2019), employs a systematic “toxic component–gene–disease” network model to characterize the toxicological profile of a substance and predict its potential toxicity through network analysis, thereby clarifying the relationship between components and adverse effects in the organism. Compared with traditional toxicology, this approach offers significant advantages including rapid assessment, resource efficiency, and high practical applicability (Liao et al., 2022). Serum sampling is minimally invasive and holds strong potential for clinical translation. Non-targeted metabolomics enables the unbiased detection of hundreds of small-molecule metabolites (Li et al., 2021), offering high sensitivity to identify early diagnostic biomarkers (e.g., lactate and ceramides), dynamically monitor the injury progression, and systematically uncover metabolic network disruptions—such as energy failure, oxidative stress, and neuroinflammation—in rats with CIRI (Figure 1).

**FIGURE 1:**
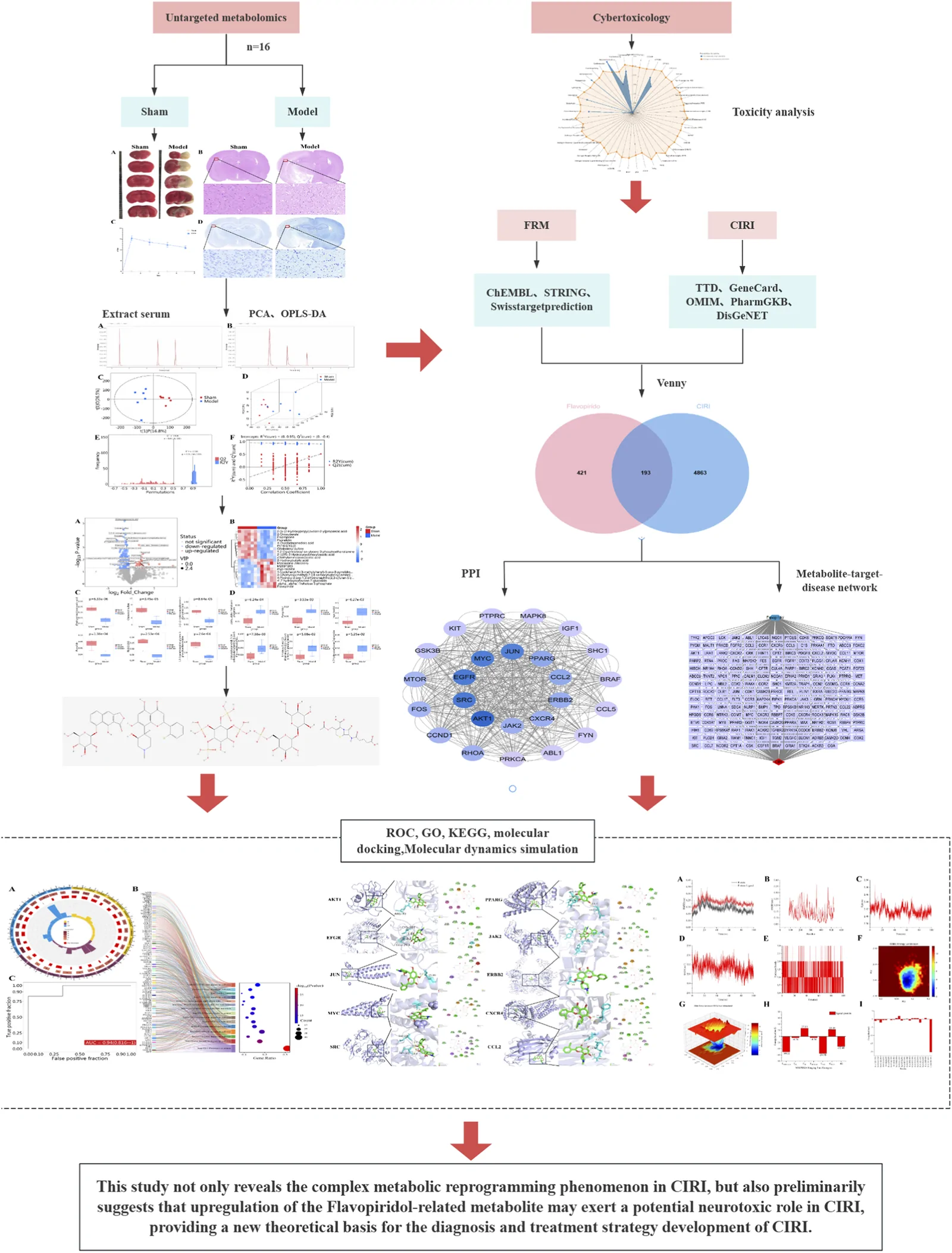
Experimental flow chart.

## 2 Materials and methods

### 2.1 Experimental animals

Sixteen specific pathogen-free (SPF) male Sprague-Dawley (SD) rats, weighing 260 ± 30 g at the start of the experiment, were supplied by Hunan Slake Jingda Co., Ltd., (Laboratory Animal License No.: 430727251100099041). After excluding animals that did not meet the modeling criteria, 12 rats were ultimately included in the study. All experimental procedures complied with the National Institutes of Health (NIH) Guide for the Care and Use of Laboratory Animals and the Animal Protection Law of the People’s Republic of China (1986), and were approved by the Institutional Animal Ethics Committee (Approval No.: SLBH-202412230002). The animals were housed under controlled environmental conditions, including a temperature of 18 °C–25 °C, relative humidity of 35%–55%, and a 12-h light/dark cycle.

#### 2.1.1 Experimental consumables and instruments

Experimental reagents: methanol (CNW Technologies, 67-56-1), acetonitrile (CNW Technologies, 75-05-8), isopropanol (CNW Technologies, 67-63-0), acetone (Sinopharm, 10000418), anhydrous ethanol (Sinopharm, 10009218), TTC staining solution (Sigma, T8877), hematoxylin staining solution (ZLI-9610), and eosin staining solution (ZLI-9613).

Vanquish ultra-high performance liquid chromatograph coupled with an Orbitrap Exploris 120 high-resolution mass spectrometer (Thermo Fisher Scientific), a JXFSTPRP-24 homogenizer (Shanghai Jingxin), an LGJ-10C freeze dryer (Sihuan Furui Instruments), a PS-60AL sonicator (Shenzhen Redbond), and a high-speed cryogenic tissue grinder (Servicebio, KZ-5F-3D). JB-P5 embedding machine (Junjie, Wuhan) and NIKON ECLIPSE E100 upright optical microscope (Nikon, Japan) were used. All instruments were calibrated before use.

#### 2.1.2 Experimental methods

##### 2.1.2.1 Animal grouping, randomization, and blinding

A total of 16 SPF-grade male Sprague-Dawley (SD) rats were initially purchased and acclimatized for 3 days prior to the experiment. Animals were randomly divided into a sham-operated group (Sham) and a model group (Model) using a random number table method, with 8 rats in each group. The randomization procedure was performed by a researcher not involved in the subsequent surgical procedures or data analysis. Group allocation information was concealed in sealed opaque envelopes and was not revealed until the day of surgery to ensure allocation concealment.

To minimize bias, all surgical procedures, outcome assessments, and data analyses were conducted by investigators blinded to group allocation. Specifically: the surgeon was informed of group assignment only immediately before surgery; neurological deficit scores were evaluated by two independent investigators blinded to group allocation, and the average score was used for analysis; histological evaluations (TTC, HE, and Nissl staining) and subsequent metabolomic data analysis were performed by researchers blinded to treatment group assignment. The blinding code was not broken until all data were collected and analyzed.

##### 2.1.2.2 MCAO model establishment

The modeling procedure was performed according to a previously described method (Song et al., 2025): after 12 h of fasting (with free access to water), the rats were anesthetized by intraperitoneal injection of 1% pentobarbital. The left common carotid artery (CCA), internal carotid artery (ICA), and external carotid artery (ECA) were bluntly dissected. A MCAO suture (2636-A4, Beijing Xinong Technology Co., Ltd.) was inserted via the ECA to occlude the middle cerebral artery (MCA). Insertion was halted when the black marker on the suture encountered resistance at the junction of the ECA and CCA. The suture was maintained in position for 90 min to achieve occlusion, after which it was gently withdrawn to allow reperfusion. The surgical incision was carefully sutured, and the operative area was disinfected with iodine. Throughout the procedure and until recovery of consciousness, the rats were placed on a heating pad to maintain body temperature at 37 °C–37.5 °C.

In the model group, right middle cerebral artery occlusion (MCAO) was established using the modified Longa intraluminal suture method, while the sham-operated group underwent the same surgical procedure without suture insertion. Neurological assessments, including the modified Neurological Severity Score (mNSS) and the Zea Longa score, were performed 24 h after surgery. Brain tissue was collected after seven consecutive days of drug administration.

##### 2.1.2.3 MCAO model validation and exclusion criteria

To ensure model homogeneity and data reliability, pre-established exclusion criteria were defined prior to the experiment. Animals meeting any of the following criteria were excluded from the study: ① death during or after surgery; ② failure to meet the model success criteria based on neurological function scores at 24 h post-surgery; ③ absence of infarct area confirmed by TTC staining; ④ severe complications (such as subarachnoid hemorrhage) or body weight loss exceeding 20% of initial body weight.

Neurological deficit assessment: Neurological impairment was evaluated using the mNSS system on days 1, 3, 5, and 7 post-surgery. The score ranges from 0 to 18 (0: no deficit; 1–6: mild deficit; 7–12: moderate deficit; 13–18: severe deficit). Simultaneously, Zea Longa scoring was performed (0: no deficit; 1: flexion of forelimb; 2: decreased resistance to lateral push; 3: circling to the contralateral side; 4: consciousness disturbance). The model success criteria were defined as: mNSS score ≥4 and Zea Longa score between 1 and 3. All scores were assessed by two independent investigators blinded to group allocation, and the average values were used for analysis.

TTC staining: Rats were euthanized 7 days after surgery, and whole brains were collected and sectioned coronally into 5 consecutive slices (thickness: 2 mm). Brain slices were incubated in 2% TTC solution at 37 °C for 30 min in the dark (with turning halfway through), followed by fixation in 4% paraformaldehyde for 24 h. Images were captured and infarct volume was analyzed using ImageJ software. Infarct volume (%) was calculated as: (ischemic volume of the affected side/total volume of the normal brain) × 100%. TTC staining results were also used to validate model success, and animals without infarct areas were excluded.

Hematoxylin-eosin (HE) and toluidine blue (Nissl staining): Whole brains were fixed in 4% paraformaldehyde, dehydrated through a graded alcohol series, cleared in xylene, and embedded in paraffin. Sections (4 μm thickness) were prepared and stained with HE for histopathological examination and with Nissl staining for neuronal damage assessment. Pathomorphological changes and neuronal injury in the ischemic cerebral cortex were observed under a light microscope. Staining evaluations were also performed by researchers blinded to group allocation.

##### 2.1.2.4 Final sample inclusion

Based on the above exclusion criteria, a total of 4 out of the initially enrolled 16 rats were excluded (3 due to unsuccessful modeling and 1 due to intraoperative death), resulting in a final sample size of 12 animals (n = 6 per group) for subsequent metabolomic analysis. All excluded animals were completely removed from the analysis, and no statistical methods were applied to handle missing data.

##### 2.1.2.5 Serum preparation

Rats were anesthetized by intraperitoneal injection of 2% sodium pentobarbital (40 mg/kg), and whole blood was collected from the abdominal aorta. Blood samples were allowed to stand at room temperature for 2 h, then centrifuged at 3,500 r/min for 15 min to separate serum. Serum was inactivated in a 56 °C water bath for 30 min and finally stored at −80 °C in an ultra-low temperature freezer until use.

### 2.2 Non-targeted metabolomics detection and analysis

#### 2.2.1 Serum preparation

A 200 μL aliquot of serum was mixed with 20 μL of 2 mol/L hydrochloric acid and sonicated in an ice-water bath for 5 min. Then, 780 μL of acetonitrile was added, and the mixture was vortexed thoroughly. The sample was placed in a −40 °C freezer for 30 min, followed by centrifugation at 13,800 rpm for 15 min at 4 °C. The supernatant was concentrated under a nitrogen stream and reconstituted in 80 μL of extraction solvent (methanol:acetonitrile:water = 2:2:1, containing isotope-labeled internal standards). Prior to injection, the sample was filtered through a 0.22 μm microporous membrane.

For high-throughput processing, an automated sample preparation system (Starlid™ workstation) was employed. Exactly 100 μL of serum was transferred into 400 μL of prechilled extraction solution (methanol:acetonitrile = 1:1, containing isotopic internal standards) and lysed on a 96-well protein precipitation plate at 750 rpm for 5 min. The filtrate was collected after ice-cold precipitation. To ensure analytical reliability, equal volumes of supernatant from each sample were pooled to generate quality control (QC) samples for monitoring instrument stability throughout the sequence.

#### 2.2.2 Metabolite identification and annotation workflow

##### 2.2.2.1 Chromatography and mass spectrometry conditions

An ultra-performance liquid chromatography-tandem mass spectrometry (UPLC-MS/MS) system was employed for metabolite separation and detection.

Chromatographic conditions: Metabolite separation was performed using a Vanquish UPLC system (Thermo Fisher Scientific) equipped with a Waters ACQUITY UPLC BEH Amide column (2.1 mm × 50 mm, 1.7 μm) for polar metabolite separation (Ricciotti and FitzGerald, 2011). Mobile phase A consisted of an aqueous solution containing 25 mmol/L ammonium acetate and 25 mmol/L ammonium hydroxide, while mobile phase B was acetonitrile. The sample tray temperature was maintained at 4 °C, and the injection volume was 2 μL. Elution parameters are presented in Table 1 (liquid phase elution parameters), with an injection volume of 2 μL and a column temperature of 4 °C.

**TABLE 1:** Liquid phase elution parameters.

NO	Time	Flow (mL/min)	%B
1	0	Run	
2	0	0.5	95
3	0.25	0.5	95
4	3.5	0.5	65
5	4	0.5	40
6	4.5	0.5	40
7	4.55	0.5	95
8	6	0.5	95
9	6	Stop run	

Mass spectrometry conditions: An Orbitrap Exploris 120 mass spectrometer (Thermo Fisher Scientific) was used for MS and MS/MS data acquisition under the control of Xcalibur software (version 4.4). The main parameters were as follows: sheath gas flow rate: 50 Arb; auxiliary gas flow rate: 15 Arb; capillary temperature: 320 °C; full MS resolution: 60,000; MS/MS resolution: 15,000; collision energy: SNCE 20/30/40; spray voltage: 3.8 kV (positive ion mode) or −3.4 kV (negative ion mode).

##### 2.2.2.2 Data processing and metabolite identification

Raw data were converted to mzXML format using ProteoWizard software. Metabolite identification was performed using an R package collaboratively developed by our laboratory. This program performed database searching against the BiotreeDB (V3.0) database (Park et al., 2006) based on accurate mass (mass error <5 ppm) and MS/MS spectral matching. BiotreeDB is a commercial metabolomics database containing accurate mass, MS/MS spectra, and retention time information for over 10,000 endogenous and exogenous metabolites. Successful matching criteria were defined as: mass error <5 ppm and MS/MS spectral similarity score >0.7 (based on dot product algorithm).

##### 2.2.2.3 Metabolite confidence level classification

To ensure transparency and traceability of identification results, all detected features were systematically classified according to the metabolomics identification confidence levels proposed by the Metabolomics Standards Initiative (MSI). The raw data comprised 3 quality control (QC) samples and 18 experimental samples, from which a total of 52,027 features were extracted. Following data preprocessing (missing value filtering and normalization):

Level 1: Metabolites validated with reference standards (retention time, MS1 accurate mass, and MS/MS spectra all matching those of the reference standard). A total of 3 such metabolites were identified in this study, primarily used for system stability and retention time calibration.

Level 2: Metabolites matched based on MS/MS spectra against databases (with clear MS/MS fragmentation information and spectral similarity score >0.7 against the BiotreeDB reference spectra). A total of 212 such metabolites were identified in this study, including the key differential metabolites reported herein.

Level 3: Metabolites putatively annotated based solely on accurate mass and/or database prediction (based on MS1 accurate mass and database retrieval but lacking reliable MS/MS spectral confirmation). A total of 1,988 such metabolites were identified in this study, serving as candidate metabolites for subsequent screening.

Level 4: Unknown compounds (unmatched to any database or lacking characteristic fragmentation information). A total of 49,196 such features were identified in this study and were not included in biological interpretation.

#### 2.2.3 Data analysis and processing

The raw data were subjected to peak alignment, baseline correction, and normalization, and then imported into SIMCA software (V18.0.1, Sartorius Stedim Data Analytics AB, Umea, Sweden). Logarithmic (LOG) transformation and centering (CTR) scaling were applied for data formatting, followed by automatic modeling analysis (Wiklund et al., 2008). Principal component analysis (PCA) and orthogonal partial least squares-discriminant analysis (OPLS-DA) were performed. The predictive ability of the models was evaluated using 7-fold cross-validation, and the validity of the models was further assessed through permutation testing.

For metabolite identification, MS/MS spectra were matched against the Human Metabolome Database (Wishart et al., 2022) (HMDB, https://hmdb.ca/), the MassBank of North America (MoNA, https://mona.fiehnlab.ucdavis.edu/), and the NIST20 spectral library, with metabolites showing a match score >80% being retained. Student’s t-test was used to calculate raw p-values for comparisons between the two groups, followed by Benjamini–Hochberg false discovery rate (FDR) correction. Metabolites that satisfied both VIP >1 and FDR-corrected q-value <0.05 were considered significantly differential metabolites.

For functional enrichment analysis, the Database for Annotation, Visualization, and Integrated Discovery (DAVID) (Sherman et al., 2022) was used to perform hypergeometric distribution tests to identify enriched pathways and functional terms associated with the differential metabolites.

#### 2.2.4 Metabolite ROC curve analysis

The raw peak areas acquired from nontargeted liquid chromatography–mass spectrometry (LC–MS) were normalized using Pareto scaling. Receiver operating characteristic (ROC) analysis was performed with MedCalc software (version 20.1), in which metabolite relative abundance served as the independent variable and group assignment as the dependent variable. The area under the ROC curve (AUC) and its 95% confidence interval were computed via the DeLong method. The optimal cutoff value was selected by maximizing the Youden index, and the corresponding sensitivity and specificity were reported. A two-sided significance level of α = 0.05 was applied for all statistical tests.

### 2.3 Network toxicology analysis

#### 2.3.1 Database and website

This study integrated the metabolite information databases such as Pubchem (Kim et al., 2023) (https://pubchem.ncbi.nlm.nih.gov/), ChEMBL (Zdrazil et al., 2024) (https://www.ebi.ac.uk/chembl/), ADMETlab 2.0 https://admetmesh.scbdd.com/, ProTox-3.0 (https://tox.charite.de/, GeneCard (Stelzer et al., 2016) https://www.genecards.org/), Therapeutic Target Database (Zhou et al., 2024) (TTD, http://db.idrblab.net/ttd/), Online Mendelian Inheritance in Man (Amberger et al., 2015) (OMIM, https://omim.org), PharmGKB (Whirl‐Carrillo et al., 2021) (https://www.pharmgkb.org/), DisGeNET (Piñero et al., 2019) (https://www.disgenet.org) for complete and systematic metabolite toxicology analysis, and used databases such as STRINGSTRING (Szklarczyk et al., 2023) (https://cn.string-db.org/), DAVID (Sherman et al., 2022) (https://david.ncifcrf.gov/), Kyoto Encyclopedia of Genes and Genomes (KEGG) (Ogata et al., 1999), UniProt (Bateman et al., 2023) (https://www.uniprot.org/), PDB (Burley et al., 2023) (http://www.rcsb.org/) for data processing and visualization.

#### 2.3.2 Compound toxicity analysis (ProTox) and target interaction network construction

The five most significantly altered metabolites identified by untargeted metabolomics were selected as potential key biomarkers for CIRI. Their SMILES notations and molecular formulas were retrieved from the PubChem database. Organ-specific toxicity profiles, with emphasis on neurotoxicity and related endpoints, were predicted using the ProTox-3.0 platform. Molecular targets of these metabolites were screened by integrating three databases: ChEMBL, STRING, and SwissTargetPrediction. The combined results constituted a compound-target dataset for subsequent network analysis. Meanwhile, CIRI-related targets were collected from five disease databases by searching the keywords “cerebral ischemia reperfusion”, “cerebral ischaemia reperfusion”, “cerebral ischemia-reperfusion” and “cerebral ischaemia-reperfusion”.

To identify overlapping targets between toxic metabolite-related proteins and CIRI-associated genes, a Venn diagram analysis was conducted using the online bioinformatics platform (http://www.bioinformatics.com.cn). The resulting intersection targets were considered to have a high potential association with CIRI pathogenesis. Protein–protein interaction (PPI) analysis of these overlapping targets was performed using the STRING database, with a minimum interaction confidence score set at ≥0.4. The resulting network was imported into Cytoscape (Shannon et al., 2003) (V3.10.3), and node centrality was evaluated using the built-in NetworkAnalyzer plugin. Targets were ranked by degree centrality, and the top 17 metabolite-related targets were selected as core candidates for subsequent visualization and analysis.

Potential targets and differentially expressed metabolites were analyzed for GO functional annotation and KEGG pathway enrichment using the DAVID database, with a significance threshold of *P* < 0.05. To visually represent the overall regulatory mechanism through which potential toxic metabolites may influence CIRI-related pathways by acting on key targets, we integrated the above analytical results and constructed a multi-level “toxic metabolite–target–disease” interaction network using Cytoscape software.

### 2.4 Molecular docking

To predict and evaluate the binding affinity and interaction modes between the identified metabolites and potential protein targets, the two-dimensional structures of metabolites were retrieved from the PubChem database, while the three-dimensional structures of the target proteins (selected from the top 10 nodes by degree value in the PPI network) were obtained from the Protein Data Bank (PDB). Protein and ligand structures were preprocessed and visualized using PyMOL software (version 2.4.0). Molecular docking was performed using AutoDock Vina (version 1.1.2) to simulate the binding behavior of toxic metabolites to core target proteins. All docking parameters were set to the software’s default values.

### 2.5 Molecular dynamics (MD) simulation

This study performed molecular dynamics simulations to evaluate the stability and dynamic behavior of protein–ligand complexes. All simulations were carried out with GROMACS 2025 under the following conditions: the protein was described with the AMBER99SB-ILDN force field, and ligand parameters were generated using the GAFF2 force field. The complex was solvated in a cubic box with TIP3P water molecules, maintaining a minimum distance of 1.2 nm from the complex to the box boundaries. Periodic boundary conditions were applied in all directions. System neutrality and physiological ionic strength were achieved by adding Na^+^ and Cl^−^ ions at a concentration of 0.15 M. Energy minimization and system equilibration were conducted under NVT and NPT ensembles (310 K, 1 bar) for 2 ns using a coupling constant of 0.1 ps. Production simulations were run for 100 ns under the NPT ensemble (310 K, 1 bar) with a time step of 2 fs, saving trajectories every 1,000 steps for subsequent analysis. The following properties were computed using built-in GROMACS tools: root mean square deviation (RMSD), root mean square fluctuation (RMSF), radius of gyration (Rg), solvent accessible surface area (SASA), number of hydrogen bonds between protein and ligand, two- and three-dimensional free energy landscapes, and binding free energy via the MM-PBSA approach.

### 2.6 Statistical analysis

Statistical analysis was performed using GraphPad Prism version 8.0, and all data were expressed as mean ± standard deviation (±s). The analysis began with tests for normal distribution and homogeneity of variance across all groups. If the data met the criteria for normality (*P* > 0.05) and homogeneity of variance (*P* > 0.05), one-way analysis of variance (ANOVA) was subsequently applied, with *post hoc* comparisons between groups conducted using the least significant difference (LSD) method. In cases where variances were unequal, Dunnett’s T3 test was used. For data that did not follow a normal distribution, the rank sum test was employed. A P-value of <0.05 was considered to indicate a statistically significant difference between groups.

## 3 Result

### 3.1 Effects of CIRI on neurological deficit score, cerebral infarction volume and pathological damage of brain tissue in rats

The Longa score and mNSS assessment revealed that neurological deficit scores in the Model group were significantly higher than those in the Sham group on day 1 after surgery (*P* < 0.01). TTC staining demonstrated no obvious infarct area (indicated by white zones) in brain tissues of the Sham group, whereas the Model group exhibited a significant increase in cerebral infarct volume compared to the Sham group (*P* < 0.01). Histopathological evaluation via HE and Nissl staining showed that cortical neurons in the Sham group were arranged in an orderly manner, with intact cellular morphology, uniform cytoplasm, and clear round nucleoli, without signs of neuronal degeneration or necrosis. In contrast, cortical neurons in the Model group were disorganized and sparsely distributed, with a notable reduction in cell count, structural disruption, and evident nuclear pyknosis, fragmentation, and necrotic changes. These findings collectively indicate the successful establishment of the CIRI model (Figure 2).

**FIGURE 2:**
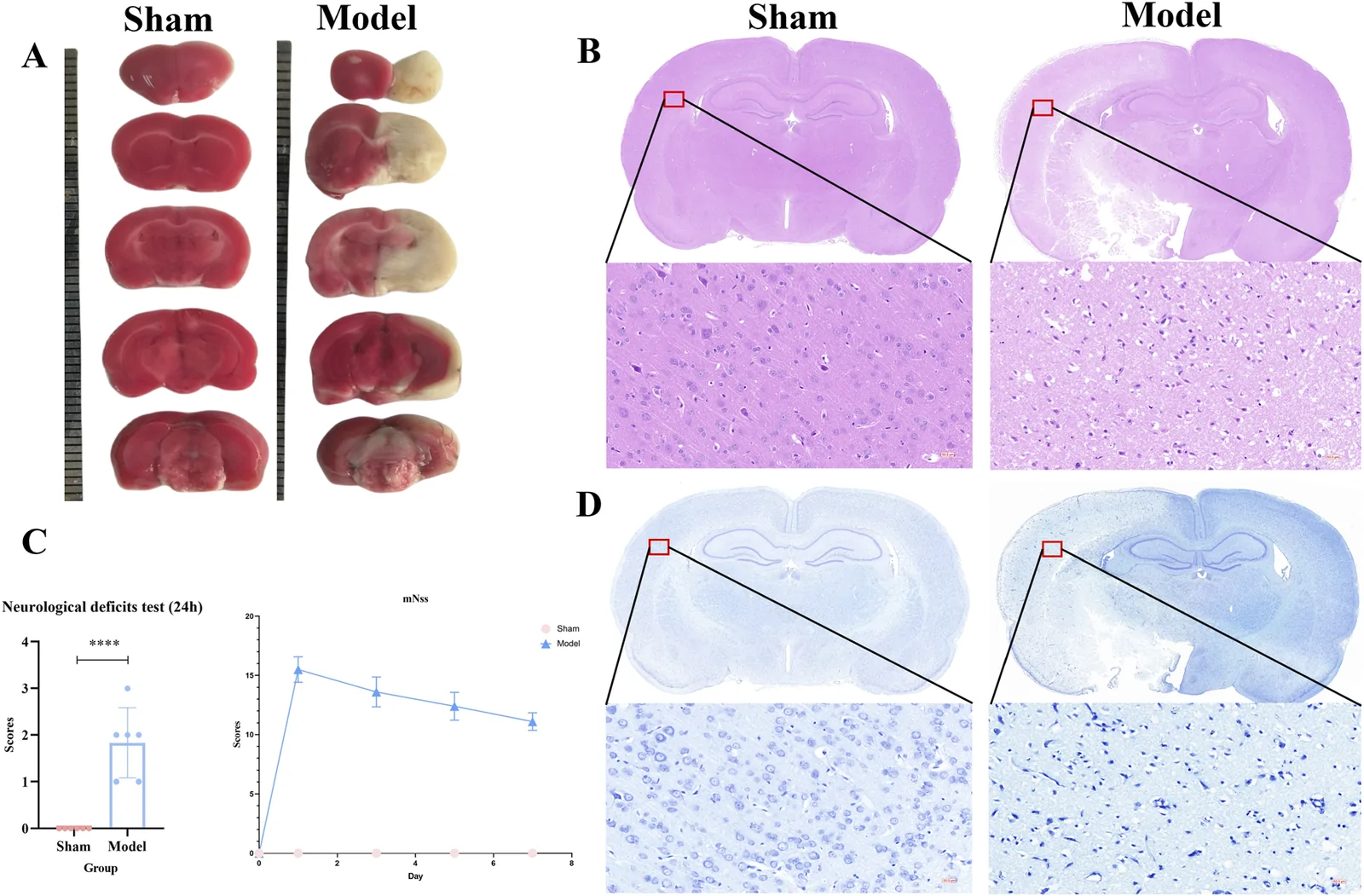
MCAO model construction. **(A)** TTC method for determination of cerebral infarction volume (****P < 0.01 vs. Sham); **(B)** HE staining of cerebral cortex (n = 3, scale bar = 50 μm); **(C)** Longa Score and Neurological Severity Score (mNSS) at 7 days Post-Modeling; **(D)** Nissl staining of cerebral cortex (n = 3, scale bar = 50 μm).

### 3.2 Metabolomics analysis

#### 3.2.1 Data quality control

The stability of the detection can be determined by the difference in the peak height of the internal standard response between quality control (QC) samples. As shown in Figures 3A,B, the retention times and peak intensities of target compounds in the QC samples exhibited high stability throughout the analytical sequence. No significant chromatographic peaks corresponding to internal standards were detected in any samples, confirming the absence of carryover contamination. PCA score plots demonstrated that sample points from both groups were predominantly distributed within the 95% confidence interval (Figures 3C,D), reflecting satisfactory data quality, minimal inter-sample contamination, and high instrumental stability, in accordance with internationally accepted metabolomics quality control guidelines (Werf et al., 2007). The OPLS-DA model further validated the metabolic profile separation between groups, with model parameters (R^2^Y = 0.903, Q^2^ = 0.516) indicating strong goodness-of-fit and predictive capability (Figures 3E,F).

**FIGURE 3:**
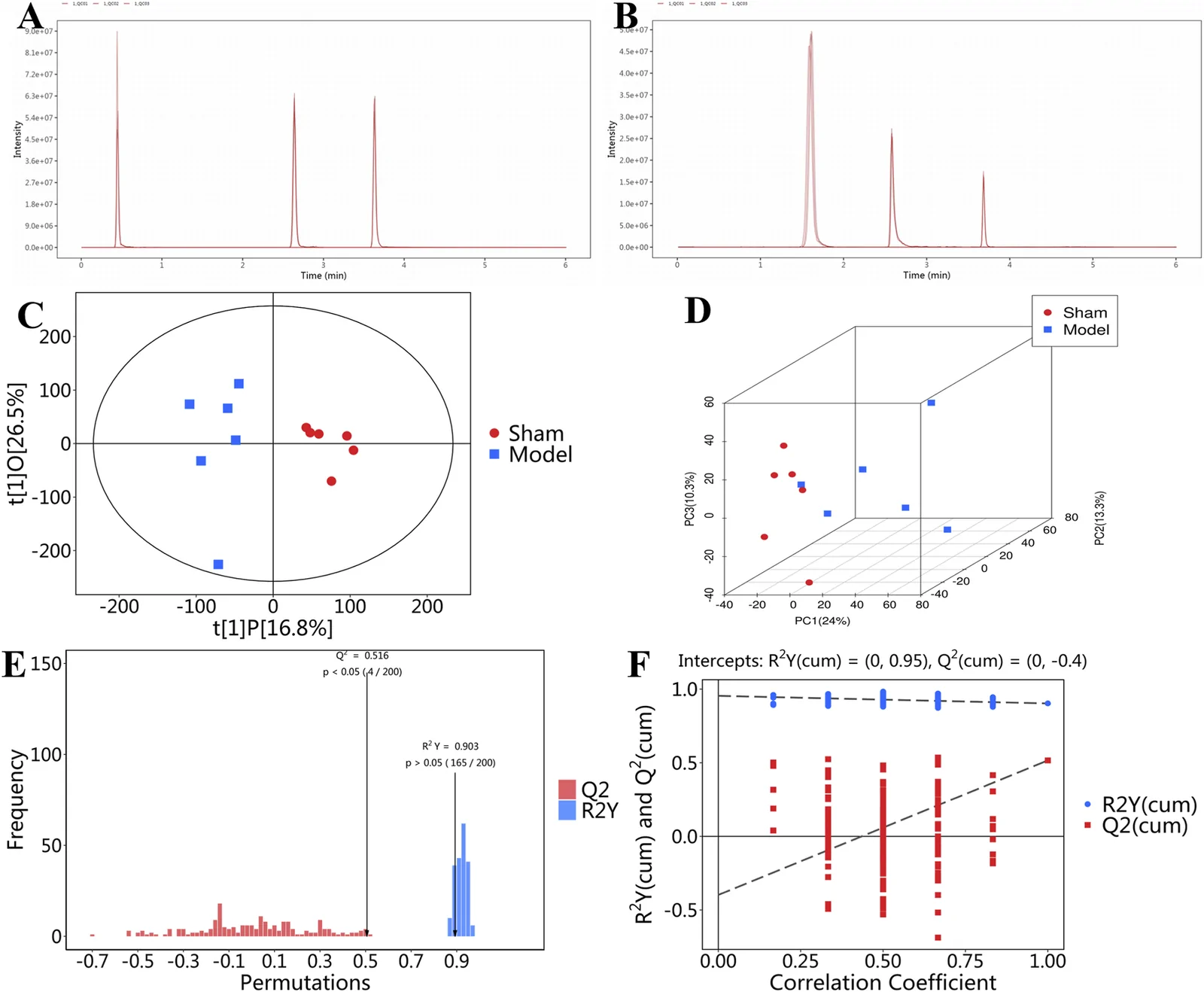
Quality control analysis. **(A,B)** EIC plots of internal standard positive and negative ions in QC samples; **(C)** Scatter plot of OPLS-DA model scores of the Model group versus the Sham group; **(D)** Scatter plot of the three-dimensional scores of the PCA model of the Model group versus the Sham group; **(E)** Dot plot of the permutation test results of the OPLS-DA model of the Model group versus the Sham group; **(F)** Histogram of the permutation test results of the OPLS-DA model of the Model group versus the Sham group.

#### 3.2.2 Differential metabolite screening

A total of 273 significantly differentially expressed metabolites were identified in serum samples from model versus sham-operated rats, based on the criteria of variable importance in projection (VIP) >1 and *P* < 0.05. Among these, 41 metabolites were significantly upregulated and 232 were downregulated (for the complete list, see Supplementary File: Table 2). According to the HMDB classification system, these differential metabolites were categorized as follows: lipids and lipid-like molecules (72, 29.4%), organic acids and derivatives (58, 23.7%), benzene and substituted benzenes (34, 13.9%), organic heterocyclic compounds (41, 16.7%), phenylpropanoids and polyketides (19, 7.8%), organic oxygen compounds (14, 5.7%), and nucleosides, nucleotides, and analogues (6, 2.4%).

To characterize the overall metabolic alterations, the top 10 most significantly up- and downregulated metabolites (by |log_2_ (fold change)|) were visualized using a volcano plot and a cluster heatmap (Figures 4A,B; detailed data in Table 2). For clearer illustration of individual metabolite changes, box plots were generated for the six most significantly altered metabolites (Figures 4C,D). Among these, α,α′-trehalose 6-phosphate, flavopiridol -related metabolite (FRM), and myo-inositol were markedly upregulated, while (diethylamino) (oxo)acetic acid, cholesteryl sulfate, and 2-[5-(2-hydroxypropyl)oxolan-2-yl]propanoic acid were significantly downregulated.

**FIGURE 4:**
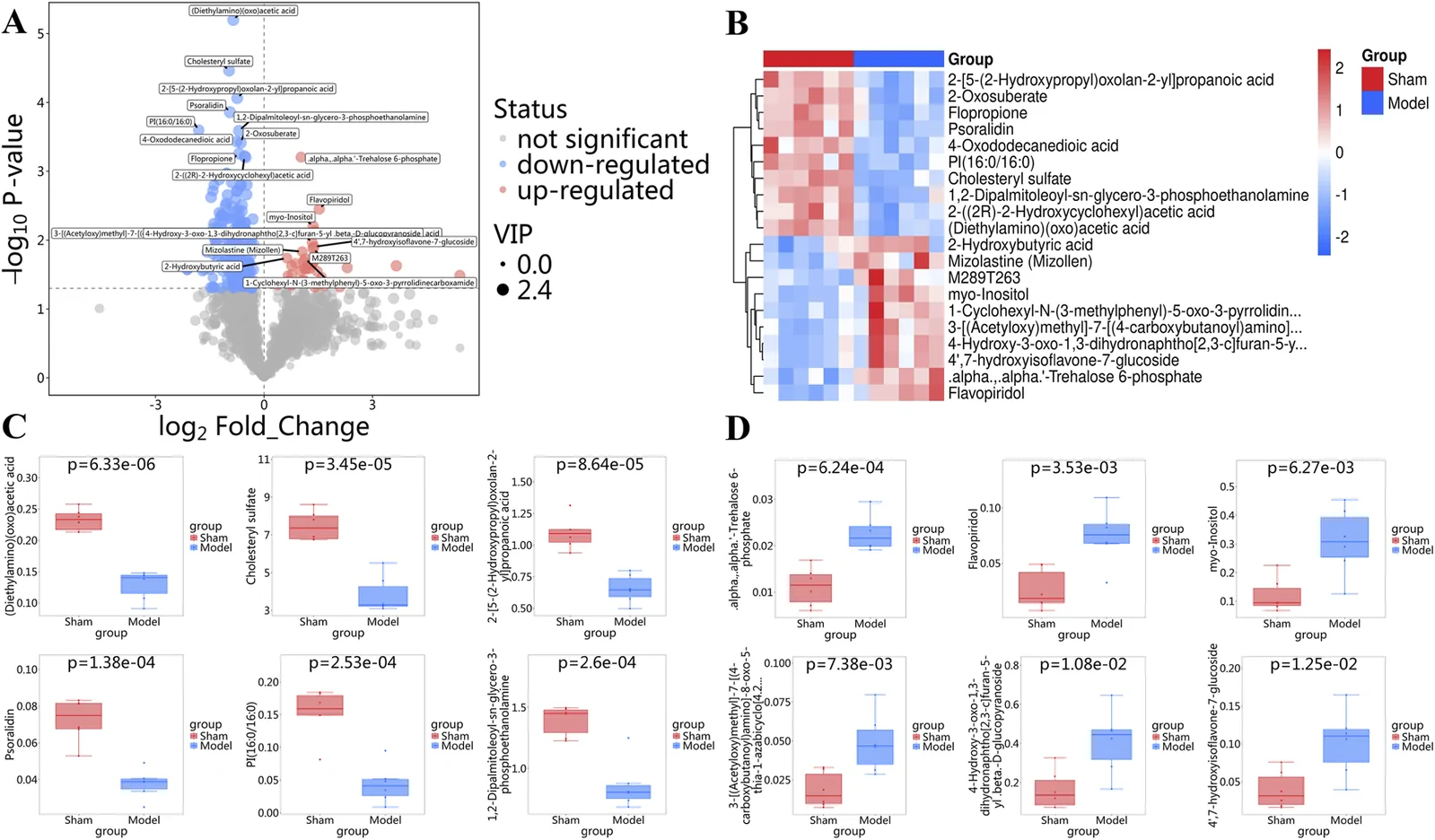
Volcano plot and cluster heat map of differential metabolites between Sham and Model. **(A,B)** Differential metabolites and heat map between Sham and Model; **(C)** Box-plot of upregulated metabolites; **(D)** Box-plot of downregulated metabolites.

**TABLE 2:** Table of changes in endogenous differential metabolites in serum of MCAO rats.

ID	MS2 name	VIP	P-value	Log_Foldchange	Dem
1	Alpha.,.alpha.'-trehalose 6-phosphate	1.655	0.00062	1.0194	Up
2	Flavopiridol	1.942	0.00353	1.5252	Up
3	Myo-inositol	1.944	0.00627	1.3537	Up
4	3-[(Acetyloxy)methyl]-7-[(4-carboxybutanoyl)amino]…	1.934	0.00738	1.3949	Up
5	4-Hydroxy-3-oxo-1,3-dihydronaphtho [2,3-c]furan-5-yl	1.795	0.01081	1.3314	Up
6	4′,7-hydroxyisoflavone-7-glucoside	1.796	0.01249	1.3755	Up
7	M289T263	2.056	0.01328	1.3518	Up
8	Mizolastine (Mizollen)	1.554	0.01469	1.0524	Up
9	2-Hydroxybutyric acid	1.620	0.01823	0.6322	Up
10	1-Cyclohexyl-N-(3-methylphenyl)-5-oxo-3-pyrrolidinecarboxamide	1.757	0.01852	1.1367	Up
1	(Diethylamino) (oxo)acetic acid	2.300	0.00001	−0.8518	Down
2	Cholesteryl sulfate	2.204	0.00003	−0.9673	Down
3	2-[5-(2-Hydroxypropyl)oxolan-2-yl]propanoic acid	2.258	0.00009	−0.7428	Down
4	Psoralidin	2.353	0.00014	−0.9426	Down
5	PI(16:0/16:0)	1.978	0.00025	−1.8069	Down
6	1,2-Dipalmitoleoyl-sn-glycero-3-phosphoethanolamine	2.225	0.00026	−0.6949	Down
7	4-Oxododecanedioic acid	2.057	0.00037	−0.7563	Down
8	2-Oxosuberate	2.215	0.00039	−0.6426	Down
9	Flopropione	2.272	0.00056	−0.6905	Down
10	2-((2R)-2-Hydroxycyclohexyl)acetic acid	2.285	0.00061	−0.5414	Down

### 3.3 Results of combined analysis of network toxicology and non-targeted metabolomics

#### 3.3.1 Toxicity analysis

Using the ProTox-3.0 database, we performed toxicity profiling on five upregulated endogenous metabolites, including α,α′-trehalose 6-phosphate, flavopiridol -related metabolite (FRM), myo-inositol, 3-[(acetyloxy)methyl]-7-[(4-carboxybutanoyl)amino]…, and 4-hydroxy-3-oxo-1,3-dihydronaphtho [2,3-c]furan-5-yl β-d-glucopyranoside (Table 3). Flavopiridol -related metabolite (FRM) was predicted to exhibit the highest toxicity level (Class IV), with prominent probabilities of neurotoxicity (0.73), nephrotoxicity (0.52), respiratory toxicity (0.97), immunotoxicity (0.85), blood–brain barrier disruption (0.74), and clinical toxicity (0.67). These toxicological profiles are closely associated with nervous system injury Figure 5B), highlighting flavopiridol -related (FRM) metabolite as a key candidate metabolite involved in drug-induced neurological damage. Figure 5A presents a schematic diagram of the compound structure of FRM, illustrating the labeled elemental symbols and chemical bonds. In the following section, we explore the molecular mechanisms through which flavopiridol -related metabolite (FRM) may contribute to CIRI. The raw mass spectra of FRM in the Sham and Model groups can be found in the Supplementary File: Image 1.

**TABLE 3:** Summary of toxicity analysis of major metabolites.

ID	Metabolites	Classification	Target	Prediction	Probability
1	Alpha., .alpha.'-…	Organ toxicity	Hepatotoxicity	Inactive	0.9
		Organ toxicity	Neurotoxicity	Inactive	0.94
		Organ toxicity	Nephrotoxicity	Active	0.73
		Organ toxicity	Respiratory toxicity	Inactive	0.74
		Organ toxicity	Cardiotoxicity	Active	0.87
		Toxicity end points	Carcinogenicity	Inactive	0.82
		Toxicity end points	Immunotoxicity	Inactive	0.94
		Toxicity end points	Mutagenicity	Inactive	0.76
		Toxicity end points	Cytotoxicity	Inactive	0.76
		Toxicity end points	BBB-barrier	Active	0.83
		Toxicity end points	Ecotoxicity	Inactive	0.63
		Toxicity end points	Clinical toxicity	Inactive	0.66
		Toxicity end points	Nutritional toxicity	Inactive	0.55
2	Flavopiridol -related metabolite (FRM)	Organ toxicity	Hepatotoxicity	Inactive	0.8
		Organ toxicity	Neurotoxicity	Active	0.73
		Organ toxicity	Nephrotoxicity	Active	0.52
		Organ toxicity	Respiratory toxicity	Active	0.97
		Organ toxicity	Cardiotoxicity	Inactive	0.78
		Toxicity end points	Carcinogenicity	Inactive	0.61
		Toxicity end points	Immunotoxicity	Active	0.85
		Toxicity end points	Mutagenicity	Inactive	0.68
		Toxicity end points	Cytotoxicity	Inactive	0.62
		Toxicity end points	BBB-barrier	Active	0.74
		Toxicity end points	Ecotoxicity	Inactive	0.52
		Toxicity end points	Clinical toxicity	Active	0.67
		Toxicity end points	Nutritional toxicity	Inactive	0.55
3	Myo-inositol	Organ toxicity	Hepatotoxicity	Inactive	0.87
		Organ toxicity	Neurotoxicity	Inactive	0.91
		Organ toxicity	Nephrotoxicity	Active	0.58
		Organ toxicity	Respiratory toxicity	Inactive	0.66
		Organ toxicity	Cardiotoxicity	Active	0.83
		Toxicity end points	Carcinogenicity	Inactive	0.55
		Toxicity end points	Immunotoxicity	Inactive	0.99
		Toxicity end points	Mutagenicity	Inactive	0.6
		Toxicity end points	Cytotoxicity	Inactive	0.84
		Toxicity end points	BBB-barrier	Active	0.61
		Toxicity end points	Ecotoxicity	Inactive	0.7
		Toxicity end points	Clinical toxicity	Inactive	0.53
		Toxicity end points	Nutritional toxicity	Inactive	0.83
4	3 [(Acetyloxy) methyl]-7-…	Organ toxicity	Hepatotoxicity	Inactive	0.7
		Organ toxicity	Neurotoxicity	Inactive	0.72
		Organ toxicity	Nephrotoxicity	Active	0.7
		Organ toxicity	Respiratory toxicity	Active	0.86
		Organ toxicity	Cardiotoxicity	Inactive	0.61
		Toxicity end points	Carcinogenicity	Inactive	0.61
		Toxicity end points	Immunotoxicity	Inactive	0.99
		Toxicity end points	Mutagenicity	Inactive	0.67
		Toxicity end points	Cytotoxicity	Inactive	0.6
		Toxicity end points	BBB-barrier	Inactive	0.99
		Toxicity end points	Ecotoxicity	Inactive	0.69
		Toxicity end points	Clinical toxicity	Active	0.71
		Toxicity end points	Nutritional toxicity	Active	0.54
5	4-Hydroxy-3-oxo-1…	Organ toxicity	Hepatotoxicity	Active	0.69
		Organ toxicity	Neurotoxicity	Active	0.87
		Organ toxicity	Nephrotoxicity	Inactive	0.9
		Organ toxicity	Respiratory toxicity	Active	0.98
		Organ toxicity	Cardiotoxicity	Inactive	0.77
		Toxicity end points	Carcinogenicity	Inactive	0.62
		Toxicity end points	Immunotoxicity	Active	0.96
		Toxicity end points	Mutagenicity	Inactive	0.97
		Toxicity end points	Cytotoxicity	Inactive	0.93
		Toxicity end points	BBB-barrier	Inactive	1
		Toxicity end points	Ecotoxicity	Active	0.73
		Toxicity end points	Clinical toxicity	Inactive	0.56
		Toxicity end points	Nutritional toxicity	Inactive	0.74

**FIGURE 5:**
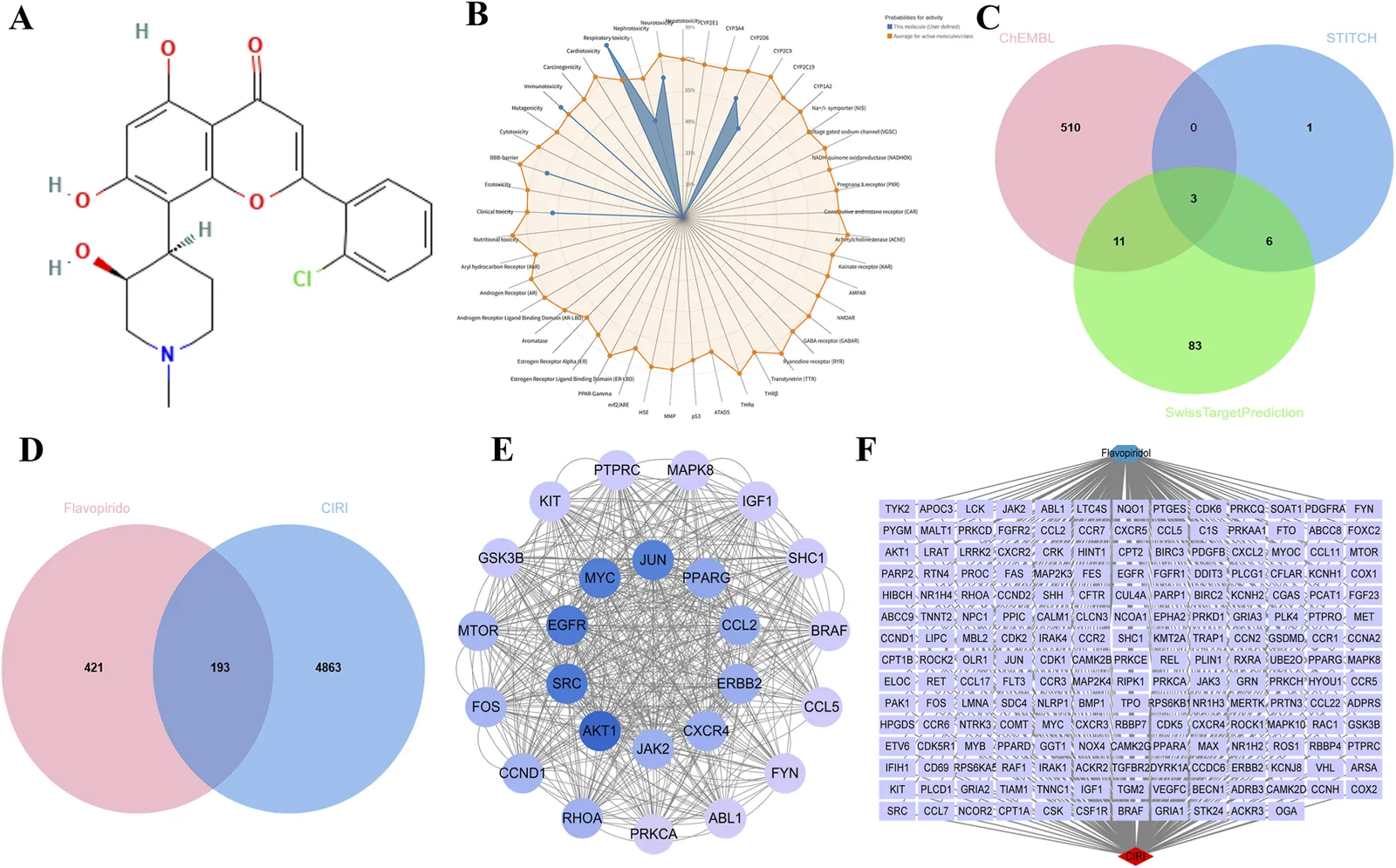
Network toxicology analysis. **(A)** 2D structure diagram of FRM; **(B)** Toxicity prediction results of FRM; **(C)** Venn diagram of the union of ChEMBL, STITCH and SwissTargetPrediction databases; **(D)** Venn diagram of the intersection targets of FRM and CIRI; **(E)** PPI network diagram; **(F)** “metabolite-target-disease” network diagram.

#### 3.3.2 FRM induced neurotoxicity and potential targets of CIRI

The ChEMBL, STITCH, and SwissTargetPrediction databases predicted 524, 10, and 110 targets for FRM, respectively. After merging and removing duplicates, a total of 614 unique targets were retained (Figure 5C). Meanwhile, 5,057 disease-associated genes related to CIRI were retrieved from five disease databases. Intersection analysis between FRM targets and CIRI-related genes identified 193 overlapping genes (Figure 5D), which were considered potential targets mediating FRM induced CIRI. A PPI network of these potential targets was constructed using the STRING database and visualized in Cytoscape. Nodes with a degree centrality ≥96 were defined as core targets (Figure 5E, where darker shading indicates higher degree). This analysis identified 10 core targets implicated in CIRI pathogenesis: AKT1, SRC, EGFR, MYC, JUN, PPARG, CCL2, ERBB2, JAK2, and CXCR4. Finally, a “metabolite–target–disease” network was generated to systematically illustrate the multi-target mechanisms underlying FRM aggravated CIRI (Figure 5F).

#### 3.3.3 Enrichment analysis and ROC analysis

GO enrichment analysis indicated that the biological processes (BP) were primarily associated with protein phosphorylation, signal transduction, inflammatory response, chemotaxis, and epidermal growth factor receptor signaling pathway. Molecular functions (MF) were mainly enriched in protein kinase activity (including serine/threonine/tyrosine kinase), ATP binding, and histone H3Y41/H2AXY142 kinase activity. Cellular components (CC) were significantly enriched in the cytosol, plasma membrane, and cell surface (Figure 6A). KEGG pathway analysis revealed that the signaling pathways were principally concentrated in core networks related to tumor development and progression, with significant enrichment in the MAPK, PI3K-Akt, Ras, Rap1, ErbB, neurotrophin, and chemokine signaling pathways, suggesting the presence of active extracellular–intracellular cascade regulatory mechanisms (Figure 6B).

**FIGURE 6:**
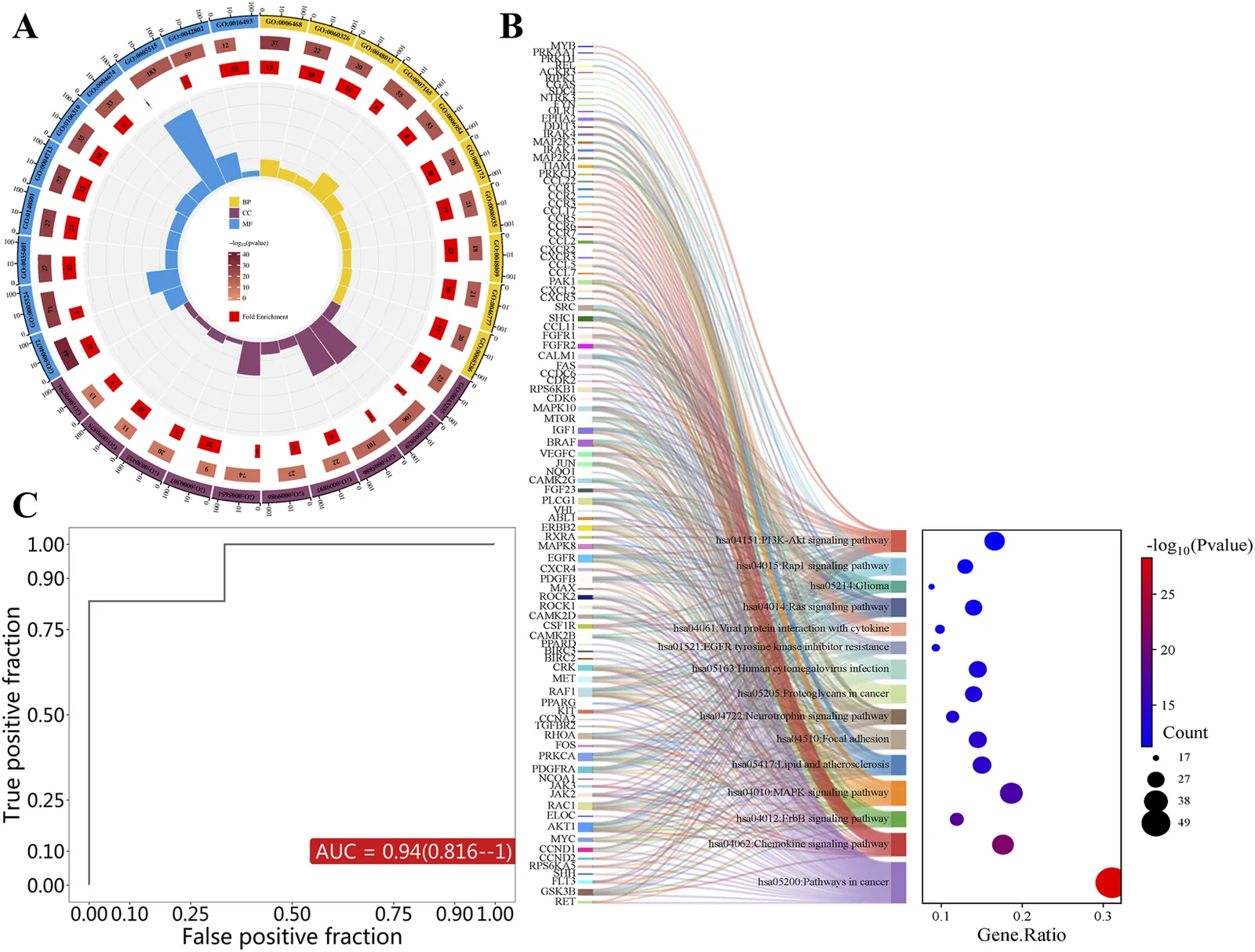
Enrichment analysis and ROC curve analysis. **(A)** GO enrichment analysis results; **(B)** KEGG enrichment analysis results; **(C)** ROC curve analysis of FRM.

Non-targeted serum metabolomics screening identified a significant upregulation of FRM following rat modeling. To assess its potential as a diagnostic biomarker, ROC curve analysis was performed based on the relative abundance of this metabolite. The results demonstrated that FRM achieved an AUC of 0.94 (95% CI: 0.816–1.000) in distinguishing the model group from the sham group, indicating high diagnostic accuracy. Both sensitivity and specificity were significant at the optimal cutoff value (Figure 6C). These findings suggest that serum FRM possesses strong discriminatory power and represents a promising metabolite marker for monitoring cerebral ischemia-reperfusion injury.

## 4 Computational biology validation

### 4.1 Molecular docking

Molecular docking results demonstrated that FRM stably binds within the active pocket of core targets in the PPI network through multiple synergistic interactions, including hydrogen bonding, π–π stacking, and hydrophobic packing, thereby effectively avoiding unfavorable steric or electronic repulsion. The calculated binding energies for all complexes were below −7 kcal·mol^-1^, indicating strong binding affinity. Notably, FRM exhibited the lowest binding energy with SRC (−9.3 kcal·mol^-1^). Structurally, the 5′-hydroxyl group of FRM forms a hydrogen bond with GLU-339, while its cyclopentane moiety deeply inserts into the hydrophobic cavity formed by MET-341, constituting a high-affinity “anchor–filler” binding mode. This structural insight provides a rational basis for optimizing SRC-targeted inhibitors—for example, by introducing additional hydrogen-bond donors toward GLU-339 or enhancing hydrophobic contact with MET-341 (Figure 7).

**FIGURE 7:**
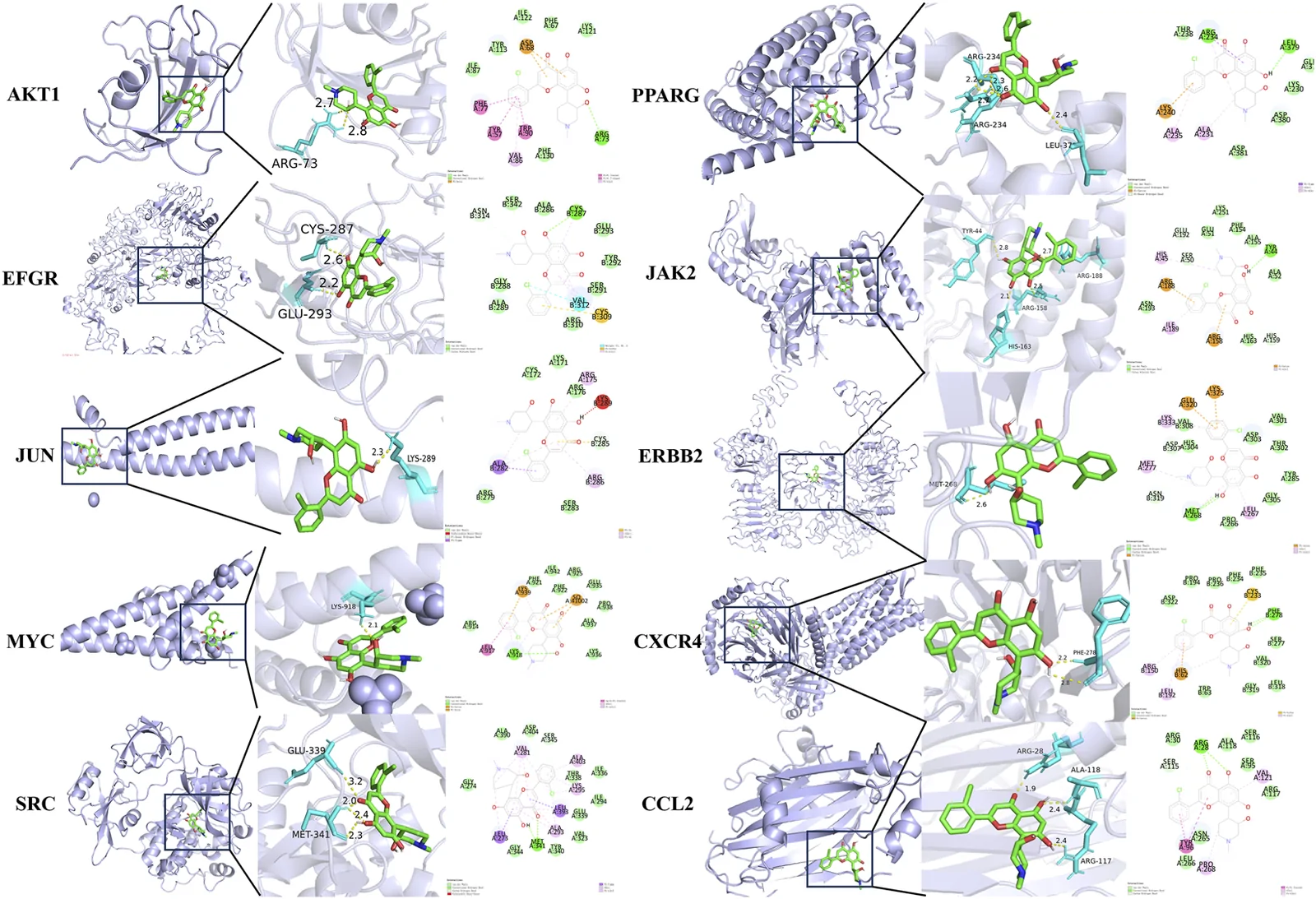
Molecular docking results. The core targets in the PPI network (AKT1, EFGR, JUN, MYC, SRC, CCL2, CXCR4, ERBB2, JAK2, and PPARG) docked with FRM, with binding energies of −8.0, −7.7, −7.3, −6.9, −9.3, −8.1, −8.8, −7.6, −7.6, and −8.0, respectively, in kcal mol^−1^.

### 4.2 Molecular dynamics simulation

In the early stages of drug discovery, molecular dynamics (MD) simulations serve as an important tool for assessing the stability of interactions between a predicted target and a drug candidate, providing preliminary computational support for target prediction and thereby aiding the efficiency of drug discovery. In this study, the FRM-SRC complex with the optimal binding energy was selected as the initial structure for MD simulations. After 10 ns of simulation, the RMSD and RMSF of the system reached equilibrium, suggesting that the protein and ligand may have achieved a relatively stable conformational state (Figures 8A,B). Analysis of the radius of gyration (rRg) and solvent-accessible surface area (SASA) further indicated that the compactness of the protein structure increased upon ligand binding, implying a potential enhancement in structural stability. The ligand remained relatively stable within the binding pocket throughout the simulation, as evidenced by the low and stable protein-ligand RMSD (P-L-RMSD) values (Figures 8C,D). Hydrogen bond analysis revealed that intermolecular hydrogen bonds may play a crucial role in the stability of the complex (Figure 8E). The binding free energy calculated over the 0–100 ns trajectory averaged −32.48 kcal/mol, indicating that the complex may possess relatively stable binding, with key residues such as LEU-393, MET-341, and GLU-339 contributing significantly to the interaction energy (Figures 8F–I). It should be emphasized that these computational results provide only theoretical support; the actual binding of FRM to SRC and their biological functions require further validation through subsequent *in vitro* binding assays and functional studies.

**FIGURE 8:**
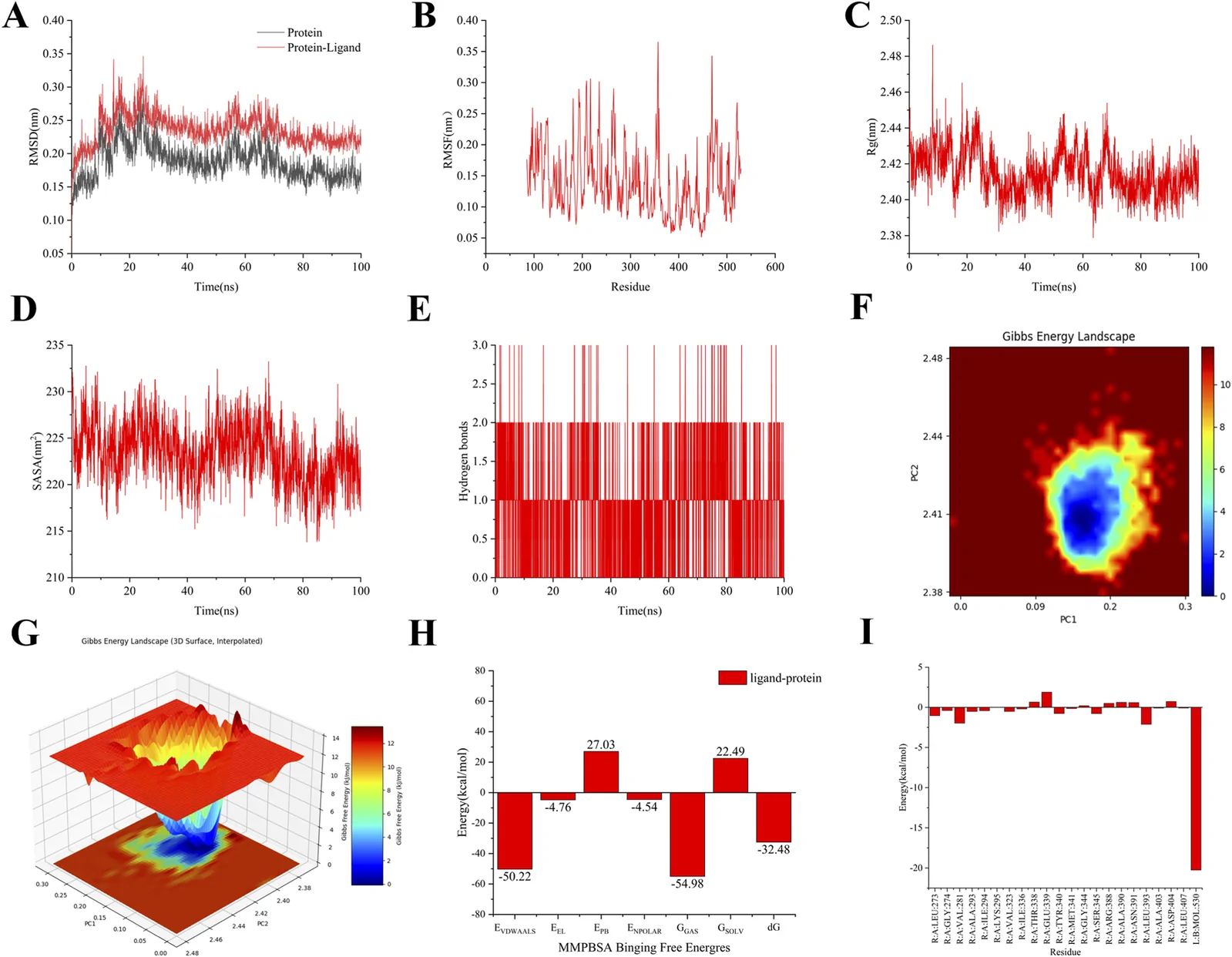
Molecular dynamics simulation results. **(A)** RMSD; **(B)** RMSF; **(C)** Rg; **(D)** SASA; **(E)** Hbond; **(F)** Gibbs 2D; **(G)** Gibbs 3D; **(H)** Ligand-protein binding free energy decomposition diagram; **(I)** Residue decomposition energy.

## 5 Discussion

In recent years, network toxicology has advanced the systematic prediction and analysis of complex interactions between toxic components and biological systems by integrating multiple public databases and bioinformatics tools, thereby enhancing our understanding of the multidimensional biological effects of toxins. This approach not only accelerates the prediction of toxicological mechanisms but also offers novel strategies for drug safety evaluation and environmental risk assessment. To investigate the mechanisms underlying toxic damage in cerebral ischemia, this study established a focal cerebral ischemia model via MCAO using a modified Longa EZ suture method. Successful induction of cerebral infarction in the model group was confirmed by mNSS neurological scores, TTC staining, HE and Nissl staining. Untargeted metabolomics analysis of post-reperfusion serum identified differentially expressed metabolites based on a threshold of VIP >1, FDR <0.05 and *P* < 0.05. Among these, metabolites such as α,α′-trehalose 6-phosphate (T6P) were significantly upregulated, while (diethylamino) (oxo)acetic acid and cholesteryl sulfate (CS) were markedly downregulated, indicating that CIRI induces substantial metabolic reprogramming. The biological significance and causal role of these metabolic shifts in brain tissue injury warrant further investigation.

As a key regulatory node in glucose metabolism, T6P directly modulates the balance between glycolysis and the pentose phosphate pathway (Liu Y. et al., 2025). Under neuronal energy stress, enhanced PPP activity accelerates the production of ribose-5-phosphate and facilitates large-scale generation of NADPH (Xu et al., 2025). As a central cellular reducing equivalent, NADPH supplies electrons to glutathione reductase, catalyzing the reduction of oxidized glutathione (GSSG) to reduced glutathione (GSH), thereby bolstering the antioxidant capacity of neurons to scavenge free radicals and mitigate reperfusion-induced lipid peroxidation, protein carbonylation, and DNA oxidative damage (Zeng et al., 2025). Moreover, T6P upregulation is closely linked to metabolic adaptation mediated by hypoxia-inducible factor-1α (HIF-1α). Under ischemic conditions, HIF-1α not only upregulates glucose transporters GLUT1/3 to enhance glucose uptake (Khiat et al., 2025) but also promotes the conversion of fructose-6-phosphate (Fru6P) to fructose-2,6-bisphosphate (F2,6BP) via transcriptional activation of PFKFB3 (Rodríguez‐García et al., 2017). As a potent allosteric activator of phosphofructokinase-1 (PFK-1), F2,6BP amplifies glycolytic flux, thereby supplying substrate for T6P synthesis. This HIF-1α-driven cascade establishes a positive feedback loop that supports redox homeostasis under metabolic stress.

As an endogenous cholesterol sulfonate derivative, CS exerts a dual vascular protective mechanism in CIRI, encompassing inflammatory regulation and blood-brain barrier (BBB) remodeling. In terms of inflammation modulation, CS activates PPARγ through isosteric activation, promoting the formation of a functional heterodimer with RXRα. This complex specifically binds to the PPRE in the promoter region of LXRα, thereby enhancing its transcriptional activity (Stelfa et al., 2025). Activated LXRα upregulates the expression of ABCA1 and ABCG1, facilitating reverse cholesterol transport and reducing pathological lipid accumulation in vascular endothelial cells. Concurrently, it physically impedes the recruitment of the NF-κB p65 subunit to inflammatory gene promoters and recruits HDAC3 to modify chromatin conformation, leading to suppression of pro-inflammatory factors such as TNF-α, IL-1β, and VCAM-1 (Wang et al., 2022). Furthermore, CS can form a charge-dependent complex with the Box B domain of HMGB1, inhibiting its interaction with RAGE and consequently blocking the downstream MAPK/NF-κB inflammatory signaling cascade (Kawakami et al., 2004). At the level of BBB maintenance, CS inhibits the catalytic activity of SGMS, reducing sphingomyelin synthesis and shifting metabolic flux toward the ceramide–sphingosine pathway (Pullmannová et al., 2014). Additionally, it allosterically activates sphingosine kinase enhancing its phosphorylation efficiency and promoting the metabolic conversion of ceramide-1-phosphate to sphingosine-1-phosphate (Wiktorek-Wójcik et al., 1997). These metabolite alterations systematically reveal profound metabolic remodeling following CIRI and elucidate disease-related mechanisms, underscoring the potential value of CS as a biomarker. Therefore, CS may serve as a novel molecular indicator for early diagnosis and disease monitoring of CIRI, providing important insights for clinical practice.

### 5.1 Analysis of the potential neurotoxicity and core target pathways of the flavopiridol-related metabolites (FRM) in CIRI

Based on untargeted metabolomics findings showing significant upregulation of endogenous metabolites after CIRI, we further employed the ProTox database to predict the toxicity of key candidate metabolites (Table 2). Notably, FRM was predicted to induce neurotoxicity, immunotoxicity, and blood–brain barrier injury—effects highly consistent with the pathophysiological features of CIRI. These toxic responses may synergistically exacerbate CIRI through several mechanisms: compromising BBB integrity, leading to peripheral inflammatory cell infiltration and aggravated cerebral edema; exerting direct neurotoxic effects that impair neuronal survival and synaptic repair; and disrupting immune homeostasis by promoting microglial polarization toward a pro-inflammatory phenotype and sustained release of damage-associated molecular patterns (DAMPs). These predictions suggest that FRM exerts multifaceted toxic effects on brain tissue. Therefore, further clarifying the neurotoxic mechanisms of FRM in CIRI through integrated network analysis and systematically identifying intervention targets for its cerebral toxicity are essential for evaluating its clinical relevance in cerebrovascular diseases and developing targeted neuroprotective strategies.

To further explore the mechanism of this metabolite in CIRI, we constructed a PPI network and performed topological analysis (screening by degree value) to identify 10 core targets interacting with FRM, such as AKT1, SRC, EGFR, JUN, and MYC. KEGG enrichment analysis further identified 15 significantly enriched signaling pathways, including PI3K-Akt and ErbB. These targets and pathways constitute a complex interactive network. Notably, the known functions of several core targets in CIRI are closely associated with the neurotoxic mechanisms predicted for FRM, suggesting its potential dual role. For instance, AKT and the PI3K/AKT pathway it regulates play a central protective role against CIRI (Liu et al., 2024), primarily by inhibiting neuronal apoptosis, excessive autophagy, and programmed necrosis through phosphorylation, while promoting neurovascular repair (Liu et al., 2023; Li et al., 2023; Liu T. et al., 2025; Zheng et al., 2024). During the secondary neuroprotection and repair phase after CIRI, the Neurovascular Unit (NVU)—composed of neurons, vascular endothelial cells, astrocytes, pericytes, and microglia—relies on the coordinated activation of intricate signaling networks, including the PI3K/Akt pathway, to facilitate the recovery of tissue homeostasis (Dai et al., 2025). However, as a potent cyclin-dependent kinase (CDK) inhibitor and transcriptional repressor, a key mechanism of FRM is its strong inhibition of the PI3K/Akt pathway (Joshi et al., 2023), which may trigger a pathological cascade and interfere with the structural and functional repair of the NVU (Zheng et al., 2024). Moreover, FRM may suppress the expression of key survival factors by inhibiting RNA polymerase II-mediated transcription, thereby reducing Akt activation at the transcriptional level (Joshi et al., 2023). Its broad-spectrum CDK inhibitory activity (e.g., against CDK2) may also indirectly affect the post-translational modification and stability of Akt (Baghel et al., 2025), thereby synergistically disrupting this pathway at multiple levels. Bioinformatics analysis suggests that FRM may attenuate intrinsic neuroprotective effects by specifically inhibiting the Akt pathway, ultimately exacerbating CIRI injury (Figures 5–7).

Similarly, elevated SRC phosphorylation levels generally exert protective effects against ischemic brain injury by regulating the proliferation and differentiation of vascular endothelial cells and promoting VEGF-mediated angiogenesis (Yu et al., 2014). However, some studies indicate that inhibiting SRC activity can suppress VEGF-induced vascular hyperpermeability (Paul et al., 2001). FRM has been shown to inhibit SRC transcription and downregulate VEGF expression (Mukhopadhyay et al., 1995), suggesting that it may disrupt neurovascular unit stability by interfering with the SRC–VEGF signaling axis. Furthermore, FRM inhibits the expression of VEGF mRNA and protein in hypoxic human monocytes by reducing VEGF mRNA stability (Melillo et al., 1999), which may represent one mechanism by which it impairs vascular repair after ischemia. Given that VEGF and its receptors are key upstream activators of the PI3K/Akt pathway, this inhibition may deprive endothelial cells and neurons of critical survival signals.

On the other hand, EGFR activation has been shown to trigger the JAK2/STAT3 pathway, exerting neuroprotective effects in CIRI (Tang et al., 2018). As a predicted target of FRM, EGFR suggests a potential mechanism by which the metabolite may interfere with survival signaling. The transcription factor c-Jun and its upstream kinase JNK significantly exacerbate neural injury by promoting neuronal apoptosis, neuroinflammation, oxidative stress, and BBB dysfunction (Johnson and Nakamura, 2007). In contrast, c-MYC, a key component of the Wnt/β-catenin pathway, has been reported to exert neuroprotective effects in CIRI, with its upregulation improving neurological function and reducing inflammation and apoptosis (Liu et al., 2022). These opposing roles highlight the complex regulatory landscape in which FRM may operate.

In summary, these targets and pathways collectively form a molecular network through which FRM may contribute to the pathogenesis of cerebral ischemia-reperfusion injury (CIRI). Our integrated analysis suggests that the CIRI-induced upregulation of these metabolites may act via upstream receptors such as EGFR and SRC, thereby perturbing multiple downstream signaling cascades, including the PI3K-Akt, MAPK, Ras, Rap1, and ErbB pathways. Such perturbations may in turn modulate the activity or expression of key transcription factors (e.g., AKT1, JUN, MYC, and PPARG) and promote the expression of CCL2, CXCR4, and their downstream kinase JAK2 (Figures 5–7). Based on this working hypothesis, we performed a preliminary evaluation of the interactions between FRM and core target proteins using molecular docking and molecular dynamics simulations. The docking results demonstrated that these metabolites exhibited strong binding affinities to all ten core targets (Figure 7), with the lowest binding energy observed for SRC (−9.3 kcal/mol), suggesting a potential interaction. Further molecular dynamics simulations revealed that the FRM-SRC complex maintained a relatively stable conformation throughout the 100 ns simulation (Figure 8), providing preliminary computational support for this interaction. It should be noted, however, that these computational findings remain theoretical predictions. The actual binding of FRM to SRC and their functional relevance in CIRI require experimental validation through subsequent *in vitro* binding assays, cellular functional studies, and animal models. Overall, the proposed molecular network and computational predictions offer a valuable hypothesis for future mechanistic studies and provide new insights into potential therapeutic strategies for CIRI.

It is important to note that the Flavopiridol-related metabolite identified in this study was not derived from endogenous sources. Flavopiridol is a synthetic compound, and its presence in rat serum is unlikely to originate from endogenous biosynthesis. Instead, the detected signal may arise from exogenous sources, such as environmental background, feed components, or potential database misannotation. Additionally, previous studies have shown that Flavopiridol can undergo metabolism in the liver to form glucuronide conjugates, which may enter the systemic circulation. Therefore, the metabolite detected in this study is more likely a xenobiotic-related compound rather than an endogenous metabolite. Nevertheless, its significant association with disease status in our statistical analysis suggests that it may serve as a valuable candidate biomarker or reflect disease-related metabolic disturbances, warranting further investigation.

### 5.2 Limitations and future directions

This study has certain limitations. Although strict model success criteria were applied during model construction (including multi-dimensional validation through neurological deficit scores, TTC staining, HE staining, and Nissl staining) and the 3R principles of animal experimentation were followed to ensure model homogeneity and data reliability, the sample size of 6 rats per group remains relatively small for non-targeted metabolomics analysis. This may affect statistical power and limit the generalizability of the findings. Therefore, the differential metabolites identified in this study should be considered preliminary candidate markers, and their robustness requires further validation with expanded sample sizes. Additionally, the proposed metabolite-target-pathway interactions are primarily based on bioinformatics predictions and computational simulations, and have not yet been functionally validated through *in vitro* or *in vivo* experiments; thus, the exact causal mechanisms remain to be confirmed in subsequent studies. Species differences between rats and humans may also impact the clinical translatability of the results.

To address these limitations, we have formulated a clear follow-up validation plan: First, mechanistic studies will be conducted at the cellular level *in vitro* to validate the specific effects of the FRM on neurovascular unit cells. Second, targeted metabolomics techniques will be employed at the animal model level to precisely quantify key metabolites, combined with biochemical indicator detection to systematically evaluate the association between its neurotoxic effects and predicted metabolic pathways. Meanwhile, key differential metabolites identified by non-targeted metabolomics will be validated using reference standards to improve the credibility of these biomarkers. These functional validation experiments will further consolidate the findings of this study and provide more robust experimental evidence for investigating the metabolic mechanisms of CIRI and identifying potential interventional targets.

## 6 Conclusion

This study integrated animal models, non-targeted metabolomics, and bioinformatics analysis to systematically investigate the metabolic alterations and potential molecular mechanisms underlying cerebral ischemia-reperfusion injury (CIRI). The focal cerebral ischemia injury model was successfully established using the middle cerebral artery occlusion method, and its stability was confirmed through multi-dimensional validation including mNSS scoring, TTC staining, HE staining, and Nissl staining. Non-targeted metabolomics analysis revealed that following CIRI, serum metabolites such as alpha, alpha'-trehalose 6-phosphate were upregulated, while (diethylamino) (oxo)acetic acid and cholesteryl sulfate were downregulated, suggesting that CIRI induces metabolic reprogramming involving multiple key pathways including energy metabolism, lipid homeostasis, neurotransmitter balance, and inflammatory oxidative stress. These disturbances collectively exacerbate the progression of secondary injury.

Further ProTox toxicity prediction and molecular network analysis indicated that the upregulated FRM- may interfere with EGFR/SRC-PI3K-Akt signal transduction, inhibit key pathways involved in neurovascular repair, and activate inflammatory-apoptotic axes such as JAK2-CCL2/CXCR4, thereby synergistically exacerbating neuronal death, neuroinflammation, and blood-brain barrier disruption. Distinct from its classical anticancer role, this study provides the first evidence suggesting that upregulation of the FRM may exert a potential neurotoxic effect in CIRI, revealing its non-classical pathological function and offering new theoretical insights into the metabolic mechanisms of CIRI and the exploration of potential therapeutic targets.

## Data Availability

The original contributions presented in the study are included in the article/Supplementary Material, further inquiries can be directed to the corresponding authors.
